# Aquatic reservoir of *Vibrio cholerae* in an African Great Lake assessed by large scale plankton sampling and ultrasensitive molecular methods

**DOI:** 10.1038/s43705-021-00023-1

**Published:** 2021-06-07

**Authors:** Luigi Vezzulli, Caterina Oliveri, Alessio Borello, Lance Gregory, Ismael Kimirei, Martina Brunetta, Rowena Stern, Simona Coco, Luca Longo, Elisa Taviani, Andrès Santos, Jaime Martinez-Urtaza, William H. Wilson, Rita R. Colwell, Carla Pruzzo, Pierre-Denis Plisnier

**Affiliations:** 1 Department of Earth, Environmental and Life Sciences (DISTAV), University of Genoa, Genoa, Italy; 2 The Marine Biological Association the Laboratory, Citadel Hill Plymouth, Devon, UK; 3 Tanzania Fisheries Research Institute (TAFIRI), Kunduchi, Dar es Salaam Tanzania; 4 Lung Cancer Unit, IRCCS Ospedale Policlinico San Martino, Genova, Italy; 5 Universidad de la Frontera, Temuco, Araucanía Chile; 6 Department of Genetics and Microbiology, Facultat de Biociéncies, Universitat Autònoma de Barcelona (UAB), Bellaterra, Barcelona Spain; 7 Maryland Pathogen Research Institute and Center of Bioinformatics and Computational Biology, University of Maryland, College Park, MD USA; 8 Johns Hopkins Bloomberg School of Public Health, Baltimore, MD USA; 9 Chemical Oceanography Unit, Institut de Physique (B5A), University of Liège, Liège, Belgium

**Keywords:** Water microbiology, Microbial ecology

## Abstract

The significance of large tropical lakes as environmental reservoirs of *Vibrio cholerae* in cholera endemic countries has yet to be established. By combining large scale plankton sampling, microbial culture and ultrasensitive molecular methods, namely Droplet Digital PCR (ddPCR) and targeted genomics, the presence of *Vibrio cholerae* was investigated in a 96,600 L volume of surface water collected on a 322 nautical mile (596 km) transect in Lake Tanganyika. *V. cholerae* was detected and identified in a large area of the lake. In contrast, toxigenic strains of V. cholerae O1 or O139 were not detected in plankton samples possibly in relation to environmental conditions of the lake ecosystem, namely very low salinity compared to marine brackish and coastal environments. This represents to our knowledge, the largest environmental study to determine the role of tropical lakes as a reservoir of *V. cholerae*.

Cholera is an acute life-threatening diarrheal disease caused by *Vibrio cholerae* serogroups O1 and O139^1^. Today, Africa is most affected by the disease, with over 41% of worldwide cholera cases and deaths reported from this continent^2^. In particular, the African Great Lake (AGL) region has suffered endemic cholera since the late 1970s, with outbreaks occurring regularly in specific districts bordering the lakes and rivers of the region^3^.

Cholera epidemiology of the AGL has been linked to introduction of toxigenic *V. cholerae* by fecal contamination of water or concurrently from environmental reservoirs of the bacteria in lake water and rivers^4,5^. The latter hypothesis, the cholera paradigm, gained attention following pioneering studies in the Bay of Bengal where the presence of *V. cholerae* in that coastal and brackish aquatic environment was linked to the incidence of cholera in neighboring villages. A strong association of the bacterium with zooplankton, namely copepods, was observed as had been demonstrated earlier in the Chesapeake Bay of the United States^6^. *V. cholerae* has been shown to require Na+ to maintain structural integrity and growth^7,8^. Nevertheless, the incidence and distribution of *V. cholerae* in tropical lake ecosystems has not been studied despite the fact that they constitute a major “hotspot” of cholera infections globally^3^. In addition, studies to date that investigated large aquatic systems as potential reservoirs of *V. cholerae* were limited in geographic extension or involved analysis of small volumes of water collected at individual point sites, hence not capturing the ecological factors associated with the disease^3^. Remarkably since these tropical areas are located in remote regions of low-income countries, accessibility to sampling sites and deployment of technologies needed to detect the presence of pathogenic bacteria in their natural reservoirs are challenging.

In this study, large scale sampling was accomplished in Lake Tanganyika and both standard bacteriological and ultrasensitive molecular methods were used to test the samples for *V. cholerae*. In total a path of 322 nautical miles (596 km) was sampled at the beginning of the short rainy season (cholera season), and 96,600 L of lake water were analysed (Fig. 1a). Sampling was conducted with the Continuous Plankton Recorder (CPR), a high-speed plankton sampler designed to be towed from ships of opportunity over long distances^9,10^ (Figs. S1 and S2). Each CPR sample represents ten nautical miles of tow (ca. 3 m^3^ of filtered water) and was previously shown to capture a substantial fraction of the plankton associated *Vibrio* community^10^.

**Fig. 1:**
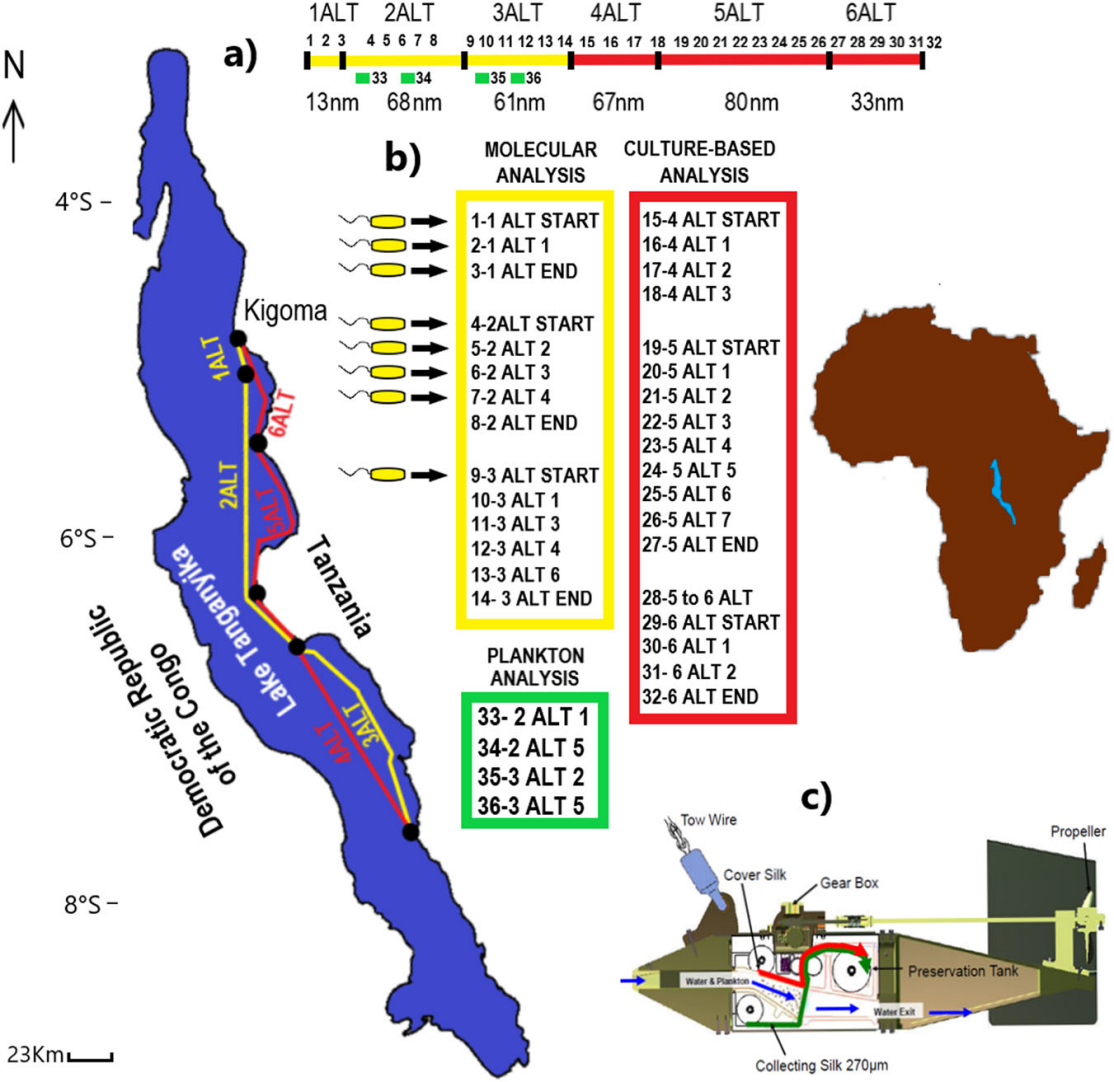
Continuous Plankton Recorder sampling in Lake Tanganyika. The sampling tows (**a**) and samples (**b**) collected in Lake Tanganyika using the Continuous Plankton Recorder (**c**). A transect of 322 nautical miles (nm) was towed October 22–26, 2018 at the beginning of the short rainy season (cholera season) with a lake water sample of 96,600 L collected and analyzed. Samples name (e.g., 1 ALT START) and number (from 1 to 36) are reported. Yellow color indicates tows and samples analyzed by molecular microbiological analysis. Red color indicates tows and samples analyzed by culture-based microbiological analysis. Green color indicate samples that were analyzed microscopically for plankton according to standard CPR procedures. Black arrows indicate samples where V. cholerae was detected using an ultrasensitive ddPCR protocol.

From October 22 to 26th 2018, six CPR tows were conducted across Lake Tanganyika (Fig. 1a–c). A total of eighteen non-formalin fixed CPR samples were collected along routes 4ALT, 5ALT and 6ALT, corresponding to ~180 nautical miles. In total ca. 54,000 L of water were analysed for the presence of *V. cholerae* by conventional culture methods^11^ (Figs. 1b and S3). A total of 27 presumptive *V. cholerae* colonies were isolated on TCBS Cholera medium and screened by *V. cholerae*-specific PCR testing and partial sequencing of the *rpoA* gene^12^. None were confirmed as *V. cholerae* (Table S1).

Since *V. cholerae* cells can be present in a viable but nonculturable (VBNC) state in environmental water, molecular analysis of the *Vibrio* community was performed on fourteen formalin fixed CPR samples collected along the 1ALT, 2ALT and 3ALT transects, corresponding to approximately 142 nautical miles, with ca. 42.000 L lake water sample collected (Fig. 1b, Fig. S4).

To detect *V. cholerae* cells with high efficiency, an ultrasensitive Droplet Digital PCR (ddPCR) protocol was developed showing a high sensitivity and robustness in detecting few genomes of *V. cholerae* (6 on ~13,000 genomes analyzed). Samples scoring positive by ddPCR were further investigated by capillary quantitative PCR assay targeting the *gpbA* (control), *ctxA, tcpA, rfbN, wbfR* genes, specifically to detect toxigenic strains^13^.

Results of the PCR analysis showed that *V. cholerae* was present in eight of the fourteen CPR samples collected over a large area of the lake (Fig. 1b) confirming that *V. cholerae* is likely present in the VBNC state in lake water (Table S2). This finding is consistent with previous reports that *V. cholerae* occurs as VBNC cells within the planktonic copepod community^14,15^. Accordingly, calanoid copepods, predominantly *Tropodiaptomus simplex*, accounted for nearly 60% of the lake plankton community (Fig. S5). Nevertheless, toxigenic *V. cholerae* O1 and O139 strains were not found. Toxigenic strains are thus lacking in pelagic waters of the lake or likely represent a very small portion of the *V. cholerae* population^15^.

To investigate the bacterial genotypes identified in the samples genome-wide enrichment of *V. cholerae* DNA from selected CPR samples (2ALTstart, 2 ALT2, and 2ALT3) was performed using hybridization-based capture employing target specific biotinylated probes (whole genome enrichment), as previously described^16^ (Fig. S4). The applied enrichment was estimated to be ca. 2500 times more effective than shotgun sequencing alone to retrieve and sequence the *V. cholerae* metagenome from complex aquatic samples^16^. By combining the targeted and shotgun metagenomic analyses, a total of 351,222,423 sequence reads (NCBI-SRA accession: PRJNA679303) were produced from the CPR samples, of which 19,886,000 reads specifically mapped against *V. cholerae* N16961 reference sequence. Taxonomic profiling and K-mer analysis of the metagenomic reads against a reference database of 466 *V. cholerae* genome sequences allowed identification of at least 10 genomic signatures belonging to non-epidemic *V. cholerae* strains (Fig. 2b). In addition, phylogenetic analysis of a reconstructed 1,017,718 nucleotide (nt) region of the metagenome-assembled genome (MAG) specifically assigned to *V. cholerae* by taxonomic binning also substantiated the presence of non-toxigenic *V. cholerae* in the samples (Fig. 2a,b), i.e., major virulence genes (e.g., *ctxAB*, *tcpA*) and epidemic markers (O1*rfb*, O139*rfb*) were not detected (Fig. 2c) (see supplementary material for more information on methods and data produced in this study).

**Fig. 2:**
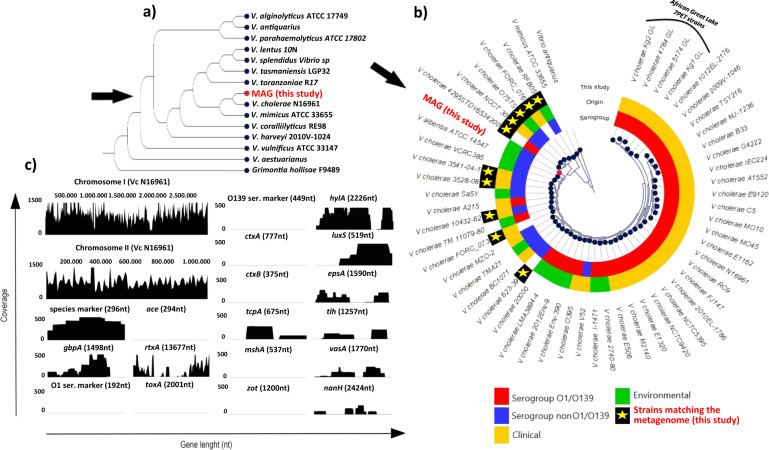
Metagenomic analysis of CPR samples. Phylogenetic analysis (**a**, **b**) of the reconstructed V. cholerae metagenome-assembled genome (MAG) sequence (indicated by arrows) based on average nucleotide identity with Vibrio reference genomes. Strain genomes (**b**) matching a 351,222,423 read sequence metagenome obtained by targeted and shotgun metagenomic analysis of CPR samples are shown (matches are indicated by yellow stars and defined by taxonomic profiling analysis of the metagenome against 466 V. cholerae genomes). **c** Read mapping analysis of the produced metagenome against the virulence factor database (http://www.mgc.ac.cn/VFs/). Only those reads uniquely mapping at a reference position were included in the analysis and further checked for specificity using BLAST against nucleotide collection (nr/nt) and RefSeq Genome databases.

In conclusion, extensive data from this study do not support the role of Lake Tanganyika pelagic water and plankton as a reservoir of *V. cholerae* strains responsible for epidemic cholera in contrast to what observed in coastal marine water and estuaries in other endemic cholera regions^6^. These findings are nevertheless consistent with studies investigating V. cholerae’s reservoirs in other freshwater bodies in Africa^17,18^. Interestingly, *V. cholerae* was detected in pelagic areas of the lake, the epidemiological relevance of which and the potential of emergence of pathogenic strains needs to be assessed^19^. That *V. cholerae* O1 or O139 toxigenic strains were not isolated appear to be linked to the different environmental conditions of Lake Tanganyika water, in particular the very low salinity (<0.4 ‰). Accordingly, a salinity of 25‰ is required for optimum growth of *V. cholerae* O1, higher than required for *V. cholerae* non O1^7^. Ecological niches for toxigenic *V cholerae* may thus only establish in confined local settings i.e. very near to the shore linked to human pollution, coastal upwelling, or episodic planktonic blooms.

## Supplementary information

Supplementary information

Supplementary data
