# Supplementary material for: Aquatic reservoir of Vibrio cholerae in an African Great Lake assessed by large scale plankton sampling and ultrasensitive molecular methods
Source: ISME Commun. 2021 Jun 7;1:20. doi: 10.1038/s43705-021-00023-1 (PMC9723668; doi:10.1038/s43705-021-00023-1)
Supplement: Supplementary file 1 — Supplementary information [file 43705_2021_23_MOESM1_ESM.pdf]

Supplementary Information for

Aquatic reservoir of *Vibrio cholerae* in an African Great Lake assessed by large scale plankton sampling and ultrasensitive molecular methods

Luigi Vezzulli[1*], Caterina Oliveri[1], Alessio Borello[1], Lance Gregory[2], Ismael Kimirei[3], Martina Brunetta[2], Rowena Stern[2], Simona Coco[4], Luca Longo[4], Elisa Taviani[1], Andrès Santos[5], Jaime Martinez-Urtaza[6], William Wilson[2], Rita R Colwell[7,8], Carla Pruzzo[1], Pierre-Denis Plisnier[9]

[1]Department of Earth, Environmental and Life Sciences (DISTAV), University of Genoa, Corso Europa 26, 16132 Genoa, Italy.
[2] The Marine Biological Association the Laboratory, Citadel Hill Plymouth, Devon PL1 2PB, UK
[3] Tanzania Fisheries Research Institute (TAFIRI), Ugweno Street, P.O.BOX 9750, Kunduchi, Dar es Salaam, Tanzania
[4] Lung Cancer Unit, IRCCS Ospedale Policlinico San Martino, Largo Rosanna Benzi, 10, 16132 Genova, Italy
[5] Universidad de la Frontera, Francisco Salazar 1145, Temuco, Araucanía, Cile
[6] Department of Genetics and Microbiology, Facultat de Biociéncies, Universitat Autònoma de Barcelona (UAB), 08193 Bellaterra, Barcelona, Spain.
[7]Maryland Pathogen Research Institute and Center of Bioinformatics and Computational Biology, University of Maryland, College Park, MD 20742
[8]Johns Hopkins Bloomberg School of Public Health, Baltimore, MD 21205
[9] Chemical Oceanography Unit, Institut de Physique (B5A), University of Liège, B-4000 Liège, Belgium

* Luigi Vezzulli
Email: luigi.vezzulli@unige.it

This PDF file includes:

Supplementary text
SI References
Supplementary Figures
Supplementary Tables

Supplementary Information Text

Material and Methods

Study area

Lake Tanganyika is situated in the western branch of the East African Rift Valley between the latitudes of 3° 30′ and 8° 50′ S and the longitudes of 29°05′ and 31°15′ E. With an approximate surface area of 32,600 km^2, Lake Tanganyika is the longest (673 km) and second deepest (1470 m) freshwater lake in the world. The volume of the lake waters (18,900 km^3) represents 17% of the Earth's surface free fresh water (1). It is shared by four countries: the Democratic Republic of the Congo (DRC), Tanzania, Zambia and Burundi. Lake Tanganyika is meromictic and oligotrophic (1,2). There is a general clockwise current in the lake (1). Meteorological conditions and particularly wind and heat exchange drive important hydrodynamics in Lake Tanganyika strongly impacting primary production (3). Some years, dense cyanobacteria blooms have been observed (4,5). Coastal planktonic blooms have also been observed from remote sensing (6). The lake waters are alkaline with a surface pH close from 9 (7) and salinity <0.5 ‰. Mean water temperature is 25°C.

In the last decades, climate change including warming and decreased winds have been noted with apparent impacts on the thermic stratification, oxygenation and primary production patterns of Lake Tanganyika (8-10).
Cholera outbreaks have occurred at Lake Tanganyika as in the East African region almost every year since 1977–1978. It has been noted that epidemics often started near the lake shores (11-12). During lull periods, persistence of cholera near the Great African lakes was explained by outbreak dynamics, which suggested a metapopulation pattern, and by endemic foci around the lakes (12). Cross analyses have indicated correlation between phytoplankton blooms and cholera outbreaks at Lake Tanganyika (13). Moore et al. (14) indicate that isolates from the African Great Lakes Region (DRC and Zambia) formed a closely related group. They also found that certain MLVA types collected in the DRC persisted in the country for several years, occasionally giving rise to expansive epidemics.

Large scale Continuous Plankton Recorder sampling in Lake Tanganyika

The CPR is a robust mechanical plankton-sampling device that has been used extensively by large merchant vessels in the oceans across the globe for more than 80 years (15) (Figure S1). For the present study, the CPR was mounted on a small transport vessel Maman Benita KAL 2271 for sampling across Lake Tanganyika. Six tows of 13 (1ALT), 68 (2ALT), 61 (3ALT), 67 (4ALT), 80 (5ALT) and 33 (6ALT) nautical miles were conducted between Kigoma and Kiranda on the eastern part of Lake Tanganyika at the beginning the short rainy season (cholera season) from 22nd to 26th October 2018. 18 standard formalin-fixed CPR samples were collected along routes 1ALT, 2ALT and 3ALT for molecular and plankton analysis. In addition, 18 non-formalin fixed CPR samples (viable-CPR protocol) were collected along routes 4ALT, 5ALT and 6ALT for culture-based microbiological studies. Sampling took place in the surface layer (~7 meters) and plankton was collected on a band of silk (mesh-size 270 μm) moving across the sampling aperture at a rate proportional to the speed of the towing ship. The CPR mesh width of 270 μm retains larger zooplankton with a high efficiency, but also collects small planktonic organisms such as nauplii, microzooplankton and phytoplankton. On return to the laboratory, the silk was removed from the device and divided into individual samples that were stored in sterile plastic boxes with or without 4% buffered formalin. Each sample represented 10 nautical miles of tow ($3m^3$ of filtered seawater) and was previously shown to capture a substantial fraction of the plankton associated *Vibrio* community (16) (Figure S1). A conductivity-temperature depth (CTD) sensor was mounted on the CPR to record surface water temperature and conductivity along the tows (Figure S2).
A key element of the CPR methodology is its ability to obtain large amounts of information from remote areas at low cost by using merchant ships of opportunity freely towing the sampling machine. The costs involved, other than for purchase of equipment, are largely for subsequent laboratory analysis of the bacteria and plankton. All samples collected during the cruise were shipped to Europe and analyzed within 14 days from collection.

***V. cholerae* culture-based studies**

Laboratory tests were initially conducted to assess recovery and culturability of *V. cholerae* strains on CPR silk under conditions mimicking those found in Lake Tanganyika environment (Figure S3). Briefly 1 ml of bacterial suspension corresponding to 10^6 cells of *V. cholerae* N16961 (ATCC 39315) strain were inoculated on $1cm^2$ sterilized CPR silk section. Silks were incubated in 5ml Eppendorf tubes containing 3ml of Artificial Seawater (ASW) or Phosphate-buffered saline (PBS) or Alkaline peptone water (APW) or unsterilized Lake water (LW) at different temperatures (+4°C and room temperature). Each condition was run in triplicate. Culturability was then assessed by conventional APW enrichment and Thiosulfate-citrate-bile salts-sucrose agar (TCBS) plating of the bacterial suspension at different time intervals of 7, 15, 28, 365 and 730 days, respectively (results showed that V. cholerae could be cultivated up to 12 weeks at room temperature on the CPR silk under all tested conditions, Figure S3).
Isolation of *V. cholerae* from CPR samples collected by the viable-CPR protocol (no formalin fixation of the CPR samples) was accomplished by employing APW enrichment of the silk (static incubation for 16h at 30 to 35°C overnight) followed by selective culture on Thiosulfate-citrate-bile salts-sucrose agar (TCBS) (incubation at 30°C to 35°C for 24 ± 2 h) as described in Huq *et al*. (17).

The silk used for enrichment corresponds to ca 3000 L of filtered lake water. Colonies grown onto TCBS agar were inspected and sucrose fermenting (yellow) colonies that have different sizes and morphologies were picked off the agar and isolated as pure cultures onto the same medium. A PCR assay (18) and partial sequencing of the *rpoA* gene (19) was used to determine if each colony belonged to the species *V. cholerae.* Table S1*.*

Nucleic acid extraction
For each CPR sample, the filtering silk was cut into strip sections (1x10 cm) and DNA was extracted and purified from each section using the methodology described in Vezzulli *et al*. (16, 18). The amount of DNA extracted from the CPR samples was determined fluorimetrically with PicoGreen using a NanoDrop® ND-3300 fluorometer (NanoDrop Technologies, Wilmington, DE, USA). Sizing of genomic DNA was also conducted in an Agilent Bioanalyzer 2100 (Agilent, Palo Alto, CA) using the High Sensitivity DNA kit (Agilent Technologies).

Droplet Digital Polymerase Chain Reaction (ddPCR) studies
Preliminary screening of CPR samples was carried out by ultrasensitive QX200 Droplet Digital PCR System (Bio-Rad Laboratories, Hercules, CA, USA) ddPCR to target the *V. cholerae* O1, O139, Non-O1, and Non-O139 specific *gbpA* marker gene (18). ddPCR is a third generation of PCR that allows absolute quantification of DNA copies by partitioning DNA single molecule into approximately 20,000 droplets based on the Poisson distribution, and then counting the number of positive and negative droplets (20). In order to estimate the ddPCR limit of detection (LoD) for detecting *V. cholerae* genomes into a sample, we firstly determined the genome mass of the pathogen using the formula m = (n) (1.096 x 10E-21 g/bp) where “m” is the genome mass in grams (1g =1E+12 pg) and n is the genome size in base pairs (bp). Therefore, since *V. cholerae* N16961 reference genome is made up of 4,033,464 bp, one haploid genome weighs 0.004421 pg. Then, we used this value to define the minimum amount of *V. cholerae* DNA that could be detected. Specifically, we made 10-fold serial dilutions starting from 0.06 ng containing only *V. cholerae* DNA to 0.00006 ng of V. *cholerae* DNA mixed with a DNA from *V. aestuarianus*, a phylogenetically close bacteria with a comparable genome size (4,837,307 bp). Briefly, DNA was mixed with Supermix no d-UTP (Bio-Rad Laboratories) and 0.4 µM primers and 0.2 µM FAM-labeled TaqMan Probe (1) in a final volume of 22 µl. Then, PCR mix was partitioned in at least 10,000 droplets by droplet generator (Bio-Rad Laboratories) and amplified in a Mastercycler nexus gradient thermal cycler (Eppendorf, Hamburg, Germany). The PCR program was as follows: enzyme activation at 95°C for 10 min followed by 40 cycles of denaturation at 94°C for 30 sec and annealing/extension at 57°C for 1 min followed by a final step of enzyme deactivation at 98°C for 10 min. Finally, the droplets were acquired into the droplet reader (Bio-Rad Laboratories), and data analyzed using the QuantaSoft software (Bio-Rad Laboratories). Notably, at a quantity of 0.00006 ng we were able to detect a mean of 6 positive droplets (two replicates) of *V. cholerae* DNA, reaching a LoD of six haploid genomes on a total of ~13,000 analyzed. Since the DNA composition in our CPR samples was unknown, we assumed that each sample contained a mix of genomes from the major subgroups of life and viruses (average genome sizes: 3,95E-02 Mb viruses; 3,214 Mb prokaryotes; 31,874 Mb fungi; 59,529 Mb unicellular eukaryotes; 855,59 Mb algae; 4456 Mb animals; 5958 Mb plants) (21) with a theoretical mean genome size of approximately 1,623 Mb (1 genome ~ 1.78 pg). In light of these considerations we used an input of 10 ng of DNA for the ddPCR, running these samples in triplicate (total DNA: 30 ng) to reach an ideal sensitivity of approximately 3 *VC* positive droplets on ~17,000 analyzed genomes. Table S2.

Capillary quantitative Real-Time PCR (qPCR) studies
CPR samples that scored positive to ddPCR were further investigated by quantitative PCR using assays targeting *gbpA* (control), *ctxA, tcpA, rfbN and wbfR* genes as described in Vezzulli *et al.* (22). Briefly, with the exception of the *gbpA* protocol (18), the LightCycler-FastStart DNA Master SYBR Green I kit (Thermofisher Scientific) optimized for use with glass capillaries and containing a hot start polymerase was used as the master mix base for all reactions. Each reaction mixture contained 5.0 mmol of MgCl2 and 500 nmol of each primer in a final volume of 20 ml. The PCR programme used was as follows: initial denaturation at 95°C for 10 min, followed by 45 cycles of denaturation at 95°C for 10 sec, annealing at 59°C for 20 sec and elongation at 72°C for 4 sec,

followed by final elongation at 72°C for 10 min. PCR runs were analyzed directly in the LightCycler using melt-curve analysis and the software provided with the instrument. The correct size of the products was further confirmed by agarose gel electrophoresis. Presence of *V. cholerae* toxigenic strains was also evaluated by Real-Time PCR using the commercial VibChoTx MONODOSE dtec-qPCR Test (Genetic PCR Solutions™, Alicante, Spain). Table S2.

Metagenomic studies

Selected CPR samples (2ALTstart, 2 ALT2 and 2ALT3) were analyzed by shotgun metagenomic and targeted metagenomic techniques (Whole Genome Enrichment) following protocols described in Vezzulli *et al* (22) (Figure S4). Briefly, genomic DNA extracted from CPR samples was used for the production of an indexed library for next-generation sequencing on the Illumina platform (Illumina, Inc) using the KAPA HyperPlus Kit for Illumina (Roche Diagnostics, Mannheim, Germany). About 200 ng of the produced shotgun metagenomic library was used for target DNA capturing using biotinylated RNA baits (on average >100-mer). Baits were produced using the MYcroarray WGE proprietary technology (MYcroarray, Ann Arbor, MI, USA) and made out from genomic DNA extracted from different *V. cholerae* strains representative of the main pathotypes: *V. cholerae* N16961 (serogroup O1, biotype El Tor), *V. cholerae* O395 (serogroup O1, biotype classical), *V. cholerae* MO10 (serogroup O139) and *V. cholerae* TMA21 (serogroup non O1/O139). The DNA library was heat-denatured and hybridized to the RNA baits under stringent conditions. Hybridization was carried out at 65°C for 36 h. After hybridization, the biotinylated baits hybridized to captured material were pulled out of the solution with streptavidin-coated magnetic beads and the captured genomic DNA was released by chemical degradation of the RNA baits. Enriched libraries were amplified and sequenced by STAB VIDA, LDA company (Caparica, Portugal) on a MiSeq Illumina™ platform (V3 flow cell, 600 cycles, 25M reads 250bp pair ends). Shotgun metagenomic libraries (not enriched libraries) were also sequenced on a Illumina® HiSeq®2500 platform (PE150 on one lane with an output of ~600M reads). Sequence reads data were archived at NCBI sequence read archive (SRA) with Accession Number PRJNA679303.

Bioinformatics analysis

Pair-read sequences from both shotgun and targeted metagenomic analyses were quality trimmed to a minimum length (75bp), with quality scores (quality nucleotide limit 0.05 based on Phred scale) and the presence of ambiguous nucleotides (n=2). Sequencing adapters were also removed. Trimmed reads were mapped against reference *V. cholerae* N16961 sequence (Accession: AE003852/AE003853) using the mapping tool of the CLC Genomics Workbench (version 20.0.4) (QIAGEN, CLC Bio, Aarthas, Denmark). A length fraction of 0.5 and similarity fraction of 0.8 were employed in the analysis (e.g. 50% minimum read length matching the reference at >80% nucleotide identity). Masking of 16SrDNA encoding genes was applied. Mapped reads-pairs obtained from shotgun and targeted metagenomic analyses for all samples were then extracted and pooled for subsequent bioinformatic analysis. Taxonomic Profiling and Find Best Matches with K-mer Spectra (Microbial Genomics Module; CLC genomics workbench) against a reference database of 466 complete *V. cholerae* genome sequences were applied for strain identification (*V.cholerae* sequences used in this study are listed in a separate excel table as supplementary). Metagenome assembly was conducted using metaSPADES (version 3.14.1) (23) and metagenome-assembled genomes (MAGs) reconstruction was carried out using CONCOCT (version 1.1.0) (24). MAGs were then refined using the CheckM (v1.1.3) 'merge' and 'outliers' tools which merge MAGs with complementary sets of marker genes to improve completeness and remove contigs from MAGs which appear to be outliers (contaminants) relative to reference GC and tetranucleotide distributions (25). MAG's taxonomic assignment to *V. cholerae* N16961 reference sequence was determined by using Kraken2 (26).

Phylogenetic analysis was performed by annotating contigs based on core genes and assigning a mass-probability to their classification with Phylosift (27). A phylogenetic tree based on alignment of the conserved codons among the strains was created with Fastree (28). Whole genome alignment and calculation of average nucleotide identity (ANI) of the MAG region with *V. cholerae* reference genomes were also applied and a tree was created through a neighbor joining clustering method with the CLC software.

Assessment of virulence genes and epidemic markers was performed by mapping reads against selected nucleotide sequences: *V. cholerae* phylogenetic marker (VC_A0047), *ace* (VFG000110), *gbpA* (VFG043577), *rtxA* (VFG000983), O1 marker region (*rfb*_region AE003852), *toxA* (VFG043673), O139 marker region (AAKF03000001), *hylA* (VFG007038), *ctxA* (VFG000107), *luxS* (VFG018241), *ctxB* (VFG000108), *epsA* (VFG040945), *tcpA* (VFG000091), *tlh* (VFG007033), *mshA* (VFG006986), *vasA* (VFG002078), *zot* (VFG000109), *nanH* (VFG001117) retrieved from the virulence factor database (29) using the mapping tool of the CLC Genomics Workbench with same settings as described above. Specificity of reads matching reference sequences was assessed by running Blastn software (version 2.10.1+; http://blast.ncbi.nlm.nih.gov/Blast.cgi) on generated consensus sequences against the nucleotide collection (nr/nt) and RefSeq Genome (refseq_genomes) database.

Sample contamination
To avoid laboratory contamination of treated samples all the analyses including DNA extraction, DNA amplification and NGS library preparations were carried out in a separate laboratory (non-aquatic/non-microbiological laboratory) using a dedicated set of pipettes, reagents, and consumables.

Plankton analysis
Qualitative assessment of plankton organisms in CPR samples (2ALT1, 2 ALT5, 3ALT2, 3ALT5) was analyzed microscopically according to standard CPR procedures as described in Reid *et al.* (30) (Figure S5).

SI References

1. Coulter, G.W. (eds) *Lake Tanganyika and its life* (Natural History Museum Publications & Oxford University Press, 1991).
2. Beauchamp R.S.A. Hydrology of Lake Tanganyika. *Int. Rev. Gesamten. Hydrobiol.* **39**, 316-353 (1939)
3. Langenberg, V.T., Sarvala, J. & Roijackers R. Effect of Wind Induced Water Movements on Nutrients, Chlorophyll-a, and Primary Production in Lake Tanganyika. *Aquatic Ecosystem Health & Management.* **6**(3), 279-288 (2003).
4. Symoens, J.J. Observation d'une fleur d'eau à Cyanophycées au Lac Tanganyika. *Folia Scient. Afr. Centr.* **1** (3): 17 (1955).
5. Salonen, K. et al. Phytoplankton in Lake Tanganyika – vertical and horizontal distribution of in vivo fluorescence. *Hydrobiologia* **407**, 89–103 (1999)
6. Plisnier, P.D. et al. Limnological variability and pelagic fish abundance (Stolothrissa tanganicae and Lates stappersii) in Lake Tanganyika. *Hydrobiologia* **625**(1), 117-134 (2009)
7. Plisnier, P.D. et al. Limnological annual cycle inferred from physical-chemical fluctuations at three stations of Lake Tanganyika. *Hydrobiologia*, 407, 45-58 (1999).
8. Plisnier, P.D. Recent climate and limnology changes in lake Tanganyika. *Verh. Internat. Verein. Limnol.* **27**, 2670-2673 (2000).
9. O'Reilly C.M., Alin, S.R., Plisnier, P.D., Cohen, A.S. & and McKee, B.A. Climate change decreases aquatic ecosystem productivity of Lake Tanganyika, Africa. *Nature* **424**, 766-768 (2003).
10. Verburg, P., Hecky, R.E. & Kling, H. Ecological consequences of a century of warming in Lake Tanganyika. *Science* **301**, 505-507 (2003).
11. Birmingham, M. et al. Epidemic cholera in Burundi: patterns of transmission in the Great Rift Valley Lake region. *The Lancet* **349**(9057), 981-985 (1997).
12. Bompangue DN. et al. Dynamics of Cholera Outbreaks in Great Lakes Region of Africa, 1978–2008. Emerg. Infect. Dis. **17**(11), 2026-2034 (2011).
13. Plisnier, P.D. et al. Cholera outbreaks at Lake Tanganyika induced by Climate Change? - "CHOLTIC". Final Report. Brussels: Belgian Science Policy 117 p. (Research Programme Science for a Sustainable Development, 2015)

14. Moore, S. et al. Relationship between distinct African cholera epidemics revealed via MLVA haplotyping of 337 Vibrio cholerae isolates. *PLoS Negl. Trop. Dis.* **9**(6), e0003817 (2015)
15. Richardson, A.J. et al. Using continuous plankton recorder data. *Prog Oceanogr* **68**, 27–74 (2006).
16. Vezzulli, L. et al. Climate influence on Vibrio and associated human diseases during the past half-century in the coastal North Atlantic. *Proc. Natl. Acad. Sci. USA 2016* **113**, E5062–E5071 (2016).
17. Huq, A. et al. Detection, isolation, and identification of *Vibrio cholerae* from the environment. *Curr. Protoc. Microbiol.* CHAPTER: Unit6A.5. doi:10.1002/9780471729259.mc06a05s26 (2012).
18. Vezzulli, L. et al. GbpA as a novel qPCR target for the species-specific detection of *Vibrio cholerae* O1, O139, Non-O1/Non-O139 in environmental, stool, and historical continuous plankton recorder samples. s. *PLoS ONE* **10**(4), e0123983. doi:10.1371/journal.pone.0123983 (2015)
19. Thompson, F.L. et al. Phylogeny and molecular identification of vibrios on the basis of multilocus sequence analysis. *Appl. Environ. Microb.* 71: 5107–5115 (2005).
20. Hindson, B.J. et al. High-throughput droplet digital PCR system for absolute quantitation of DNA copy number. *Anal. Chem.* **83**, 8604–8610 (2011).
21. Landenmark, H.K.E., Forgan, D.H. & Cockell, C.S. An estimate of the total DNA in the biosphere. *PLoS Biol*. **13**, 1–10 (2015)
22. Vezzulli, L. et al. Whole-Genome Enrichment Provides Deep Insights into Vibrio cholerae Metagenome from an African River. *Microb Ecol.* **73**, 734–738 (2017).
23. Nurk, S., Meleshko, D., Korobeynikov, A. & Pevzner, P.A. MetaSPAdes: A new versatile metagenomic assembler. *Genome Res.* **27**: 824–834 (2017).
24. Alneberg, J. et al. Binning metagenomic contigs by coverage and composition. *Nat Methods* **11**, 1144–1146 (2014).
25. Parks, D.H. et al. CheckM: Assessing the quality of microbial genomes recovered from isolates, single cells, and metagenomes. Genome Res. **25**: 1043–1055 (2015).
26. Wood, D.E., Lu, J. & Langmead, B. Improved metagenomic analysis with Kraken 2. *Genome Biol.* **20,** 257 (2019).
27. Darling, A.E. PhyloSift: Phylogenetic analysis of genomes and metagenomes. PeerJ 2:e243 https://doi.org/10.7717/peerj.243 (2014)
28. Price, M.N., Dehal, P.S. & Arkin, A.P. FastTree 2 - Approximately maximum-likelihood trees for large alignments. PLoS ONE 5(3): e9490. https://doi.org/10.1371/journal.pone.0009490 (2010)
29. Chen, L., Xiong, Z., Sun, L., Yang, J. & Jin, Q. VFDB 2012 update: Toward the genetic diversity and molecular evolution of bacterial virulence factors. *Nucleic Acids Res.* **40**, D641–D645 (2012).
30. Reid, P.C. et al. The Continuous Plankton Recorder: Concepts and history, from Plankton Indicator to undulating recorders. *Progr Oceanogr.* **57**, 117–173 (2003).

Figure S1. The Continuous Plankton Recorder (CPR)

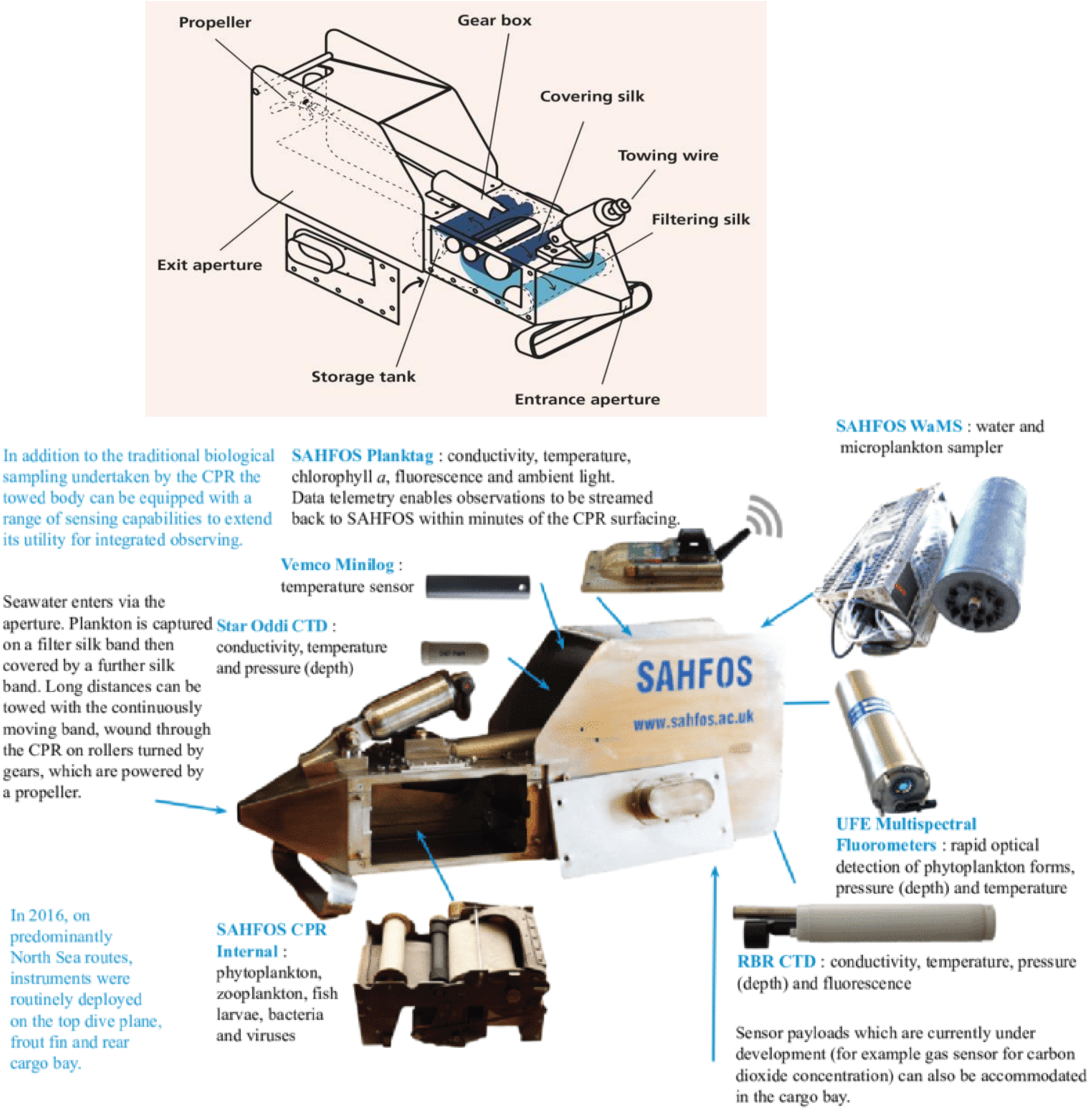

Figure S2. Water temperature profile measured by a CTD mounted on the CPR during the cruise.

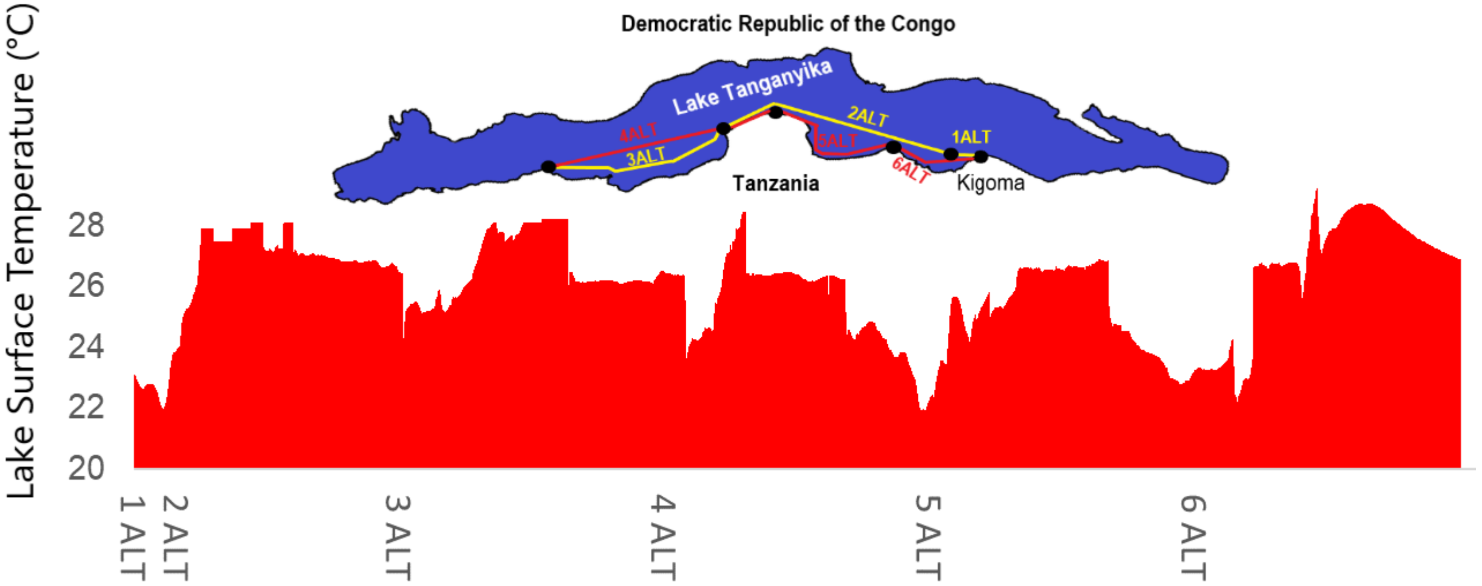

Figure S3 *V. cholerae* culturability test in CPR samples.

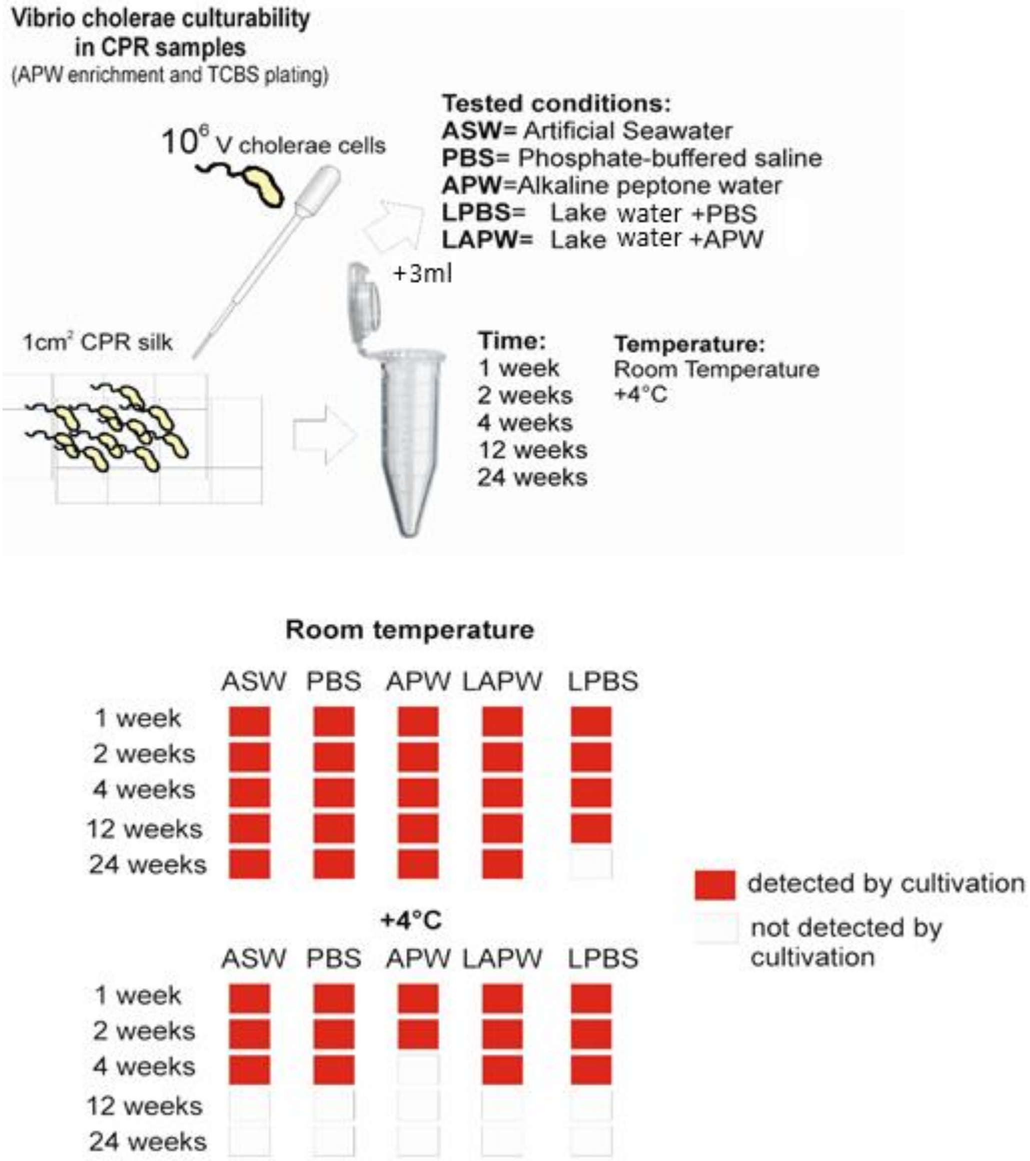

Figure S4. Metagenomic analysis workflow applied on CPR samples.

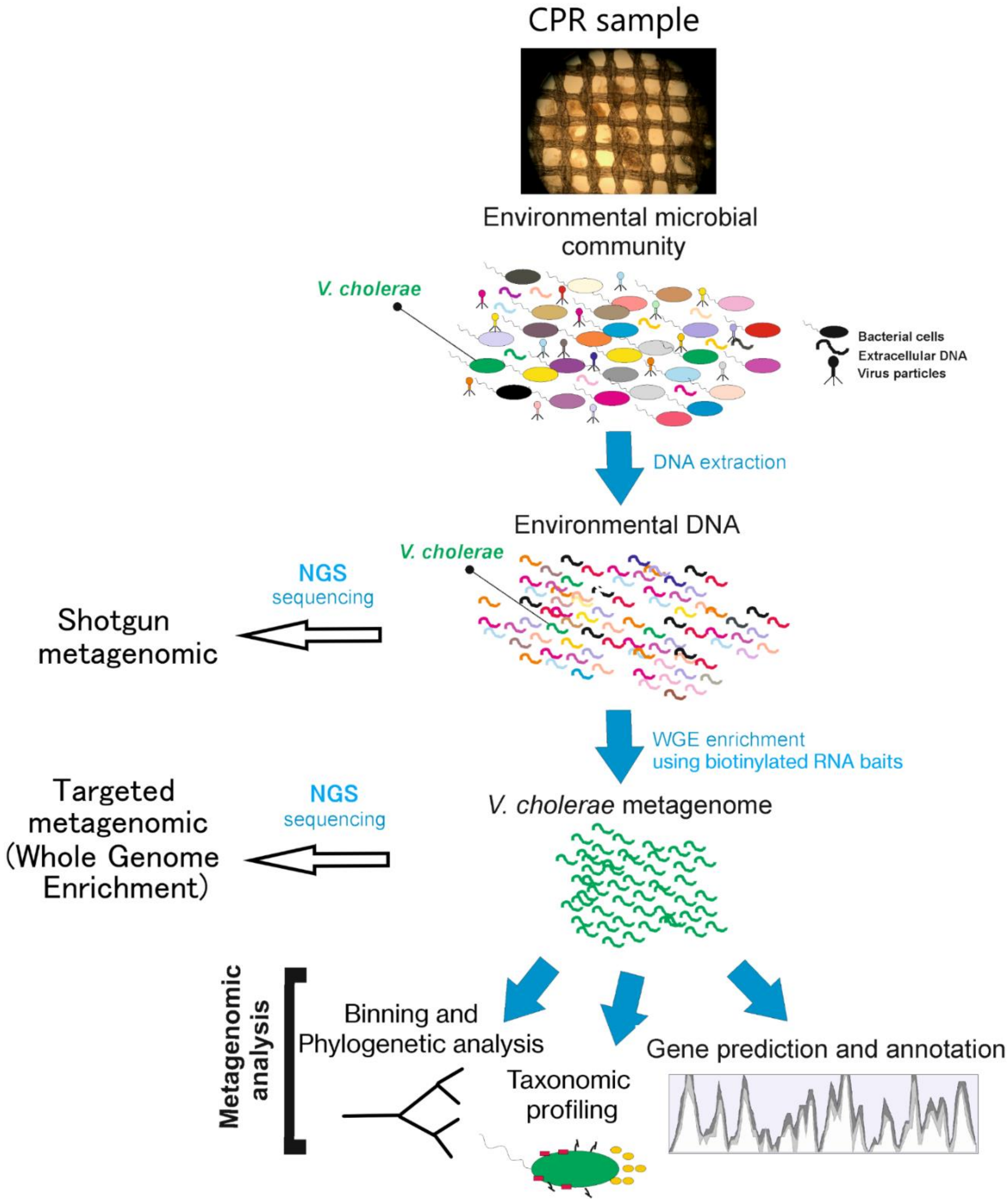

Figure S5. Average plankton composition of the collected CPR samples along routes 2ALT and 3ALT.

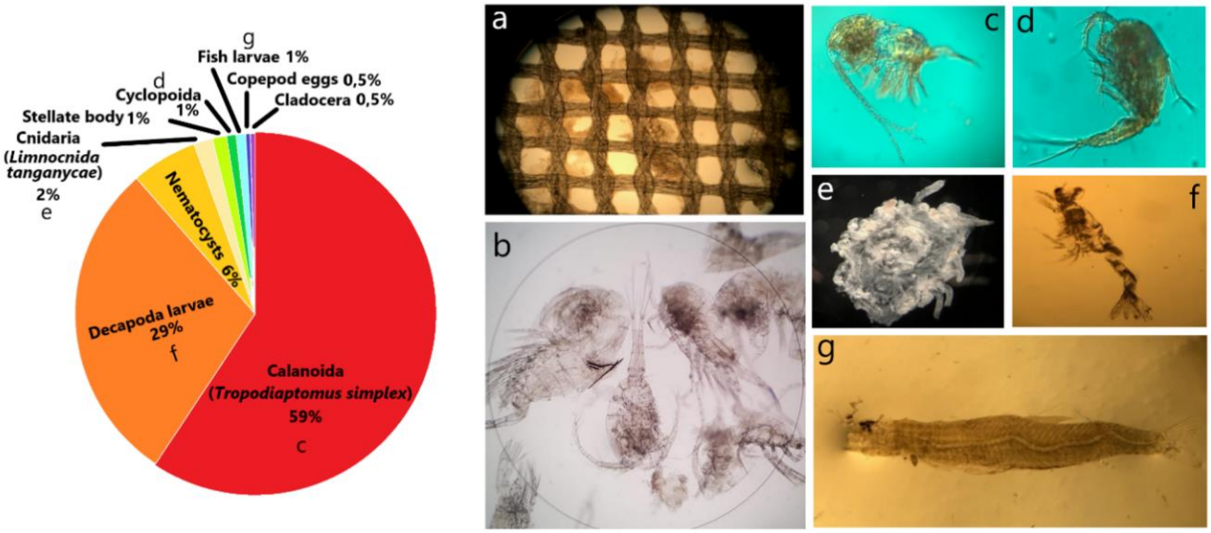

Table S1. Results of *V. cholerae* cultivation in APW and TCBS media from CPR samples collected in Lake Tanganyika

APW culture		TCBS culture						
Sample	V. cholerae PCR test	tow	morphotype	morphology	V. cholerae PCR test	rpoA sequencing Blast Best hit		rpoA sequencing Identity
4 ALT START	negative	4 ALT	1	green	negative	Leclercia sp.		100%
4 ALT 1	negative		2	small yellow	negative	Enterobacter hormaechei		100%
4 ALT 2	negative		3	yellow	negative	Enterobacter hormaechei		100%
4 ALT 3	negative							
5 ALT START	negative	5 ALT	4	yellow	negative	Aeromonas media		99,60%
5 ALT 1	negative		5	small yellow	negative	Enterobacter hormaechei		100%
5 ALT 2	negative		6	star yellow	negative	Enterobacter hormaechei		100%
5 ALT 3	negative		7	yellow with dark center	negative	Enterobacter hormaechei		100%
5 ALT 4	negative		8	yellow with salmon center	negative	Aeromonas media		99,40%
5 ALT 5	negative		9	star yellow	negative	Aeromonas hydrophila		98,50%
5 ALT 6	negative		10	salmon	negative	Aeromonas media		99,40%
5 ALT 7	negative		11	green	negative	Aeromonas veronii		98,10%
5 ALT END	negative		12	star yellow	negative	Enterobacter hormaechei		99,90%
			13	transparent yellow	negative	Enterobacter hormaechei		99,90%
			14	yellow with dark center	negative	Enterobacter hormaechei		99,70%
5 to 6 ALT	negative	6 ALT	15	star yellow	negative	Enterobacter hormaechei		99,60%
6 ALT START	negative		16	yellow with brown center	negative	Vibrio mimicus		99,80%
6 ALT 1	negative		17	yellow with dark center	negative	Vibrio mimicus		99,70%
6 ALT 2	negative		18	semitransparent yellow	negative	Enterobacter hormaechei		99,80%
6 ALT END	negative		19	yellow with salmon center	negative	Aeromonas media		99,60%
			20	yellow with dark center	negative	Enterobacter hormaechei		99,90%
			21	yellow with salmon center	negative	Aeromonas media		99,30%
			22	green	negative	Enterobacter cloacae		100%
			23	yellow	negative	Enterobacter hormaechei		99,60%
			24	yellow	negative	Enterobacter cloacae complex sp.		99,90%
			25	big yellow	negative	Enterobacter hormaechei		100%
			26	green	negative	Enterobacter cloacae complex sp.		99,90%
			27	salmon	negative	Vibrio mimicus		99,60%

Table S2 . Results of PCR and ddPCR test targeting *V. cholerae* in CPR samples collected in Lake Tanganyika (GU/rx=Genomic Unit/reaction)

Sample (positive samples)	DNA extraction (ng/ul)	Analysed DNA (ng)	n° replicate	V. cholerae qPCR (GU/rx)	Toxigenic V. cholerae qPCR (GU/rx)	V. cholerae ddPCR
1 ALT START	0,4	10	3	78,6±7,6	negative	positive
1 ALT 1	0,4	10	3	6,0±0,5	negative	positive
1 ALT END	1	10	3	5,6±0,4	negative	positive
2 ALT START	0,3	10	3	19,2±0,8	negative	positive
2 ALT 2	26,47	10	3	23,1±1,6	negative	positive
2 ALT 3	25,5	10	3	99,9±9	negative	positive
2 ALT 4	17,15	10	3	22,5±2,6	negative	positive
2 ALT END	27,42	10	3	negative	negative	negative
3 ALT START	0,5	10	3	negative	negative	positive
3 ALT 1	2,25	10	3	negative	negative	negative
3 ALT 3	5,2	10	3	negative	negative	negative
3 ALT 4	4,6	10	3	negative	negative	negative
3 ALT 6	5,33	10	3	negative	negative	negative
3 ALT END	1,55	10	3	negative	negative	negative
Control	0	10	3	negative	negative	negative
